# Using Mathematical Transmission Modelling to Investigate Drivers of Respiratory Syncytial Virus Seasonality in Children in the Philippines

**DOI:** 10.1371/journal.pone.0090094

**Published:** 2014-02-27

**Authors:** Stuart Paynter, Laith Yakob, Eric A. F. Simões, Marilla G. Lucero, Veronica Tallo, Hanna Nohynek, Robert S. Ware, Philip Weinstein, Gail Williams, Peter D. Sly

**Affiliations:** 1 School of Population Health, University of Queensland, Brisbane, Queensland, Australia; 2 University of Colorado School of Medicine, Aurora, Colorado, United States of America; 3 Research Institute for Tropical Medicine, Department of Health, Muntinlupa City, Metro Manila, Philippines; 4 Department of Vaccines and Immune Protection, National Institute for Health and Welfare, Helsinki, Finland; 5 Barbara Hardy Institute, University of South Australia, Adelaide, South Australia, Australia; 6 Queensland Children’s Medical Research Institute, University of Queensland, Brisbane, Queensland, Australia; 7 Colorado School of Public Health, University of Colorado, Aurora, Colorado, United States of America; Imperial College London, United Kingdom

## Abstract

We used a mathematical transmission model to estimate when ecological drivers of respiratory syncytial virus (RSV) transmissibility would need to act in order to produce the observed seasonality of RSV in the Philippines. We estimated that a seasonal peak in transmissibility would need to occur approximately 51 days prior to the observed peak in RSV cases (range 49 to 67 days). We then compared this estimated seasonal pattern of transmissibility to the seasonal patterns of possible ecological drivers of transmissibility: rainfall, humidity and temperature patterns, nutritional status, and school holidays. The timing of the seasonal patterns of nutritional status and rainfall were both consistent with the estimated seasonal pattern of transmissibility and these are both plausible drivers of the seasonality of RSV in this setting.

## Introduction

Acute lower respiratory infection (ALRI) is responsible for 20% of deaths in post neonatal infants globally, with one third of these deaths due to respiratory syncytial virus (RSV) [1]. In most settings ALRI in young children follows striking seasonal patterns, including in the tropics, where ALRI generally occurs during the rainy season [2], [3]. Relatively little emphasis has been placed on systematically investigating the drivers of seasonality in tropical settings, despite the fact that most ALRI deaths in children occur in the tropics. Seasonal variations in transmission are required to amplify the inherent oscillations in infection incidence, which would otherwise remain damped, and to dictate the timing of seasonal epidemics, which are generally consistent in similar climates. Possible ecological drivers of respiratory infection seasonality include increased pathogen survival (possibly due to variations in temperature, humidity or sunlight); reduced host immunity (possibly due to variations in nutrition, sunlight, or co-infections); and increased host mixing due to seasonal variations in behavioural patterns (such as increased time spent indoors during rainy or cold periods, and school terms) [4], [5]. Identifying the drivers of seasonality has potential benefit beyond improving the understanding of disease transmission. As well as improving the prediction of disease burdens in the context of global change, host factors such as poor nutrition are amenable to intervention, which may prove more effective if timed correctly [4].

In previous studies in the Philippines, we have found that seasonal variations in child nutrition, as well as in meteorological factors (rainfall, sunshine and relative humidity) were associated with the seasonality of ALRI admissions in children [6], [7]. The lag time between the seasonal peaks in these potential driving factors and the peaks in the yearly ALRI epidemics varied considerably. High rainfall and relative humidity, and low levels of sunshine all occurred within a few weeks before or after the seasonal peak in ALRI. In contrast nutritional status was poorest 10 weeks before the seasonal peak in ALRI. In this setting in the Philippines the seasonal peaks in ALRI admission are coincident with the peaks in cases of microbiologically confirmed RSV [6]. From a modelling perspective, drivers of transmission act by altering the transmission coefficient (β) which is a measure of the transmissibility of the infection (the rate at which an infectious person in the population infects susceptible people in the population) at any point in time. Note that transmissibility differs from transmission – although transmissibility may be high at a particular point in time, the number of infections actually occurring will also depend on the number of infectious and susceptible individuals at the time [4], [8]. Previous modelling suggests the lag between the yearly peak in β and the yearly peak in the incidence of infections may be in the order of one to three months for RSV infection [9]. With this in mind we have used an RSV transmission model to estimate when the seasonal scale variations in β would need to occur in order to reproduce the observed seasonality in RSV admissions in our study population. We have then compared this seasonal pattern of β to the seasonal patterns of potential environmental drivers of transmission: rainfall, humidity, and temperature patterns, nutritional status, and school holidays, in order to better assess their epidemiological plausibility.

### Ethics Statement

The trial “Effectiveness of an 11-valent pneumococcal conjugate vaccine against pneumonia in Philippine children: A double-blind, placebo-controlled, randomised, multicentre, effectiveness study” was approved and monitored by the Ethical and Institutional Review Board of the Research Institute for Tropical Medicine (RITM), the Philippines. Written informed consent was obtained from parents/guardians prior to enrolling infants into the trial. In a small minority of cases both parents were illiterate. In these instances a witness independent of the research team (a relative or neighbour) attested to (1) that the information provided to the parent was from the information sheet, (2) that the parent was given the opportunity to ask questions, and (3) that the parent provided verbal consent for the child to participate in the study. The witness then signed the consent form on behalf of the parent. The Ethical and Institutional Review Board of the RITM approved the consent protocol and information sheets. This retrospective analysis used de-identified data collected during the trial. Ethical approval for the analysis in this paper was granted without the need to seek additional consent, by the University of Queensland School of Population Health Research Ethics Committee in June 2011 (Ethics approval SP070711).

## Methods

ALRI admissions data were collected during a randomised controlled trial to investigate the efficacy of pneumococcal conjugate vaccine (PCV) in Bohol Province in the Philippines, between 2000 and 2004 [10], [11]. Study personnel were permanently assigned to hospitals involved with case ascertainment, and respiratory infections were assessed and recorded in all children living in the trial municipalities who were admitted to local health care facilities over the course of the trial. Nasal wash specimens were collected from children admitted to the Bohol Regional Hospital with ALRI, and from 1 in 3 children diagnosed with ALRI at the outpatient clinic. Children with a positive RSV polymerase chain reaction (PCR) result were included in this study.

### A. Model Structure

**Figure 1:**
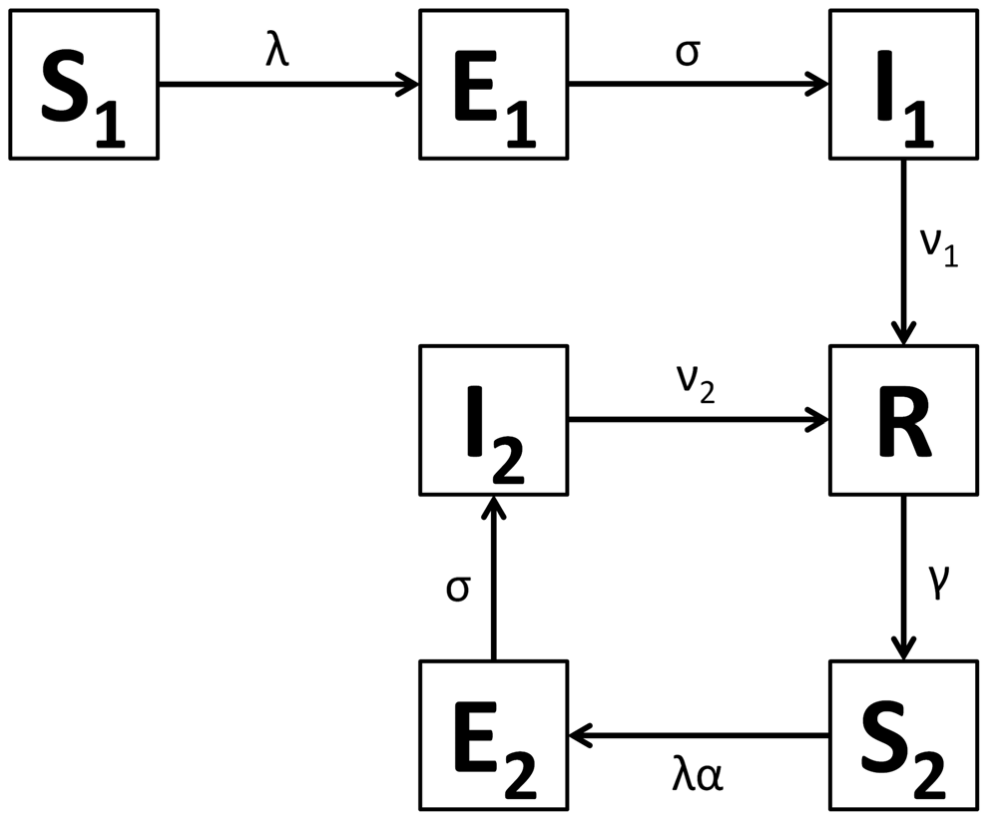
RSV model schematic. S_1_ are susceptible individuals before their first RSV infection. E_1_ are individuals infected for the first time but not yet infectious. I_1_ are individuals infected for the first time and now infectious. R are individuals recovered from infection and temporarily resistant to reinfection. S_2_ are partially susceptible individuals before later RSV infections. E_2_ are individuals with subsequent infections but not yet infectious. I_2_ are individuals with subsequent infections and now infectious.

Figure 1 shows the structure of our RSV model. The rates of movement between the different epidemiological categories are given by the following differential equations (parameter definitions are given in Table 1):
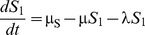


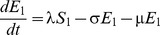


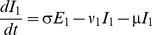


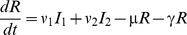


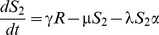


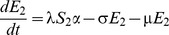


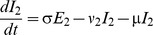



**Table 1:** Parameter estimates used in the model.

Parameter	Notation	Model estimates
Force of infection	λ	Mean = 0.0022 to 0.0037 day^−1^
Mean latent period	1/σ	4 to 6 days
Mean duration of infection in category I_1_	1/ν_1_	5 to 6 days
Mean duration of infection in category I_2_	1/ν_2_	4 days
Degree of infectiousness (I_2_/I_1_)	δ	0.5 to 0.8
Rate of loss of short term immunity (R→S_2_)	γ	0.012 to 0.024 day^−1^
Susceptibility in category S_2_	α	0.68 to 0.84

The model was integrated over daily time steps using Runge-Kutta 4 integration. The model is frequency dependent (all the model compartments sum to 1). Fully susceptible individuals (S_1_) are infected at rate λ (the force of infection) which is dependent on the transmission coefficient (β) and the proportion of infectious individuals (categories I_1_ for primary infection and I_2_ for subsequent infections):
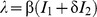



δ allows for variable transmission from subsequent infections and α allows for a variable level of susceptibility to subsequent infections when in category S_2_. For both primary and subsequent infections, there is a latent period of infection during which time individuals are exposed but not yet infectious (respectively, E_1_ and E_2_). Following the infectious period (respectively, I_1_ and I_2_), individuals move to the recovered, non-susceptible (R) category at rate ν_1_ and ν_2_ respectively. The recovered, non-susceptible state is temporary, with individuals moving from this category to the S_2_ category at rate γ. The transmission coefficient (β) takes into account host, pathogen and environmental factors that influence transmission [12].

We have modelled the seasonal variation in the transmission coefficient using a cosine function. The timing of the yearly peaks in the transmission coefficient is defined by the phase (φ) of the cosine wave:
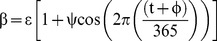



ε is the average value of β, and ψ is the magnitude of the seasonal variation (as a proportion of ε). We also examined a second version of the model with a seasonal forcing function describing a square wave with decreased transmissibility over 2 months of the year. The timing and magnitude of the square wave is determined by an underlying cosine wave:
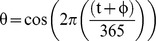


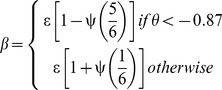



This second version of the model approximates the expected effect of school summer holidays (April–May). Although most symptomatic RSV infection occurs in infants and children too young to attend school, household studies suggest that RSV infection is often introduced into households by school aged siblings (although the proportion of household outbreaks attributed to RSV being introduced by school aged siblings varies considerably between studies) [13], [14].

Seasonality of births can also influence the seasonality of infections, in particular the seasonality of first infections [15]. We simulated this by allowing for seasonality in the birth rate, fitting a cosine wave to local birth data. Here, µ_s_ denotes the seasonal birth rate, and µ denotes the mean birth rate (set at the Philippines birth rate of 7×10^−5^ of the total population per day).
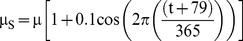



### B. Parameter Estimates

#### B1. Average force of infection (mean λ)

We have based our estimate of the force of infection on two studies measuring the incidence rate of first RSV infection over a number of years. Both studies used active case finding to identify children with respiratory infection, who were then tested for RSV infection. The first study is a follow up of two birth cohorts (n = 635) in the rural setting of Kilifi, Kenya [16]. RSV was confirmed using immunofluorescence testing of nasal specimens and/or seroconversion. The rate of first RSV infection was 0.55 (95% CI 0.50 to 0.61) per child year over the first three years of life, equivalent to a rate of 0.0022 per day. The second study is a follow up of a single birth cohort (n = 125) from the urban setting of Houston, USA [17]. This study identified RSV cases using viral culture of nasal specimens and/or seroconversion, and found a rate of first RSV infection of 0.74 (95% CI 0.62 to 0.89) per child year over the first three years of life, equivalent to a rate of 0.0037 per day.

**Figure 2:**
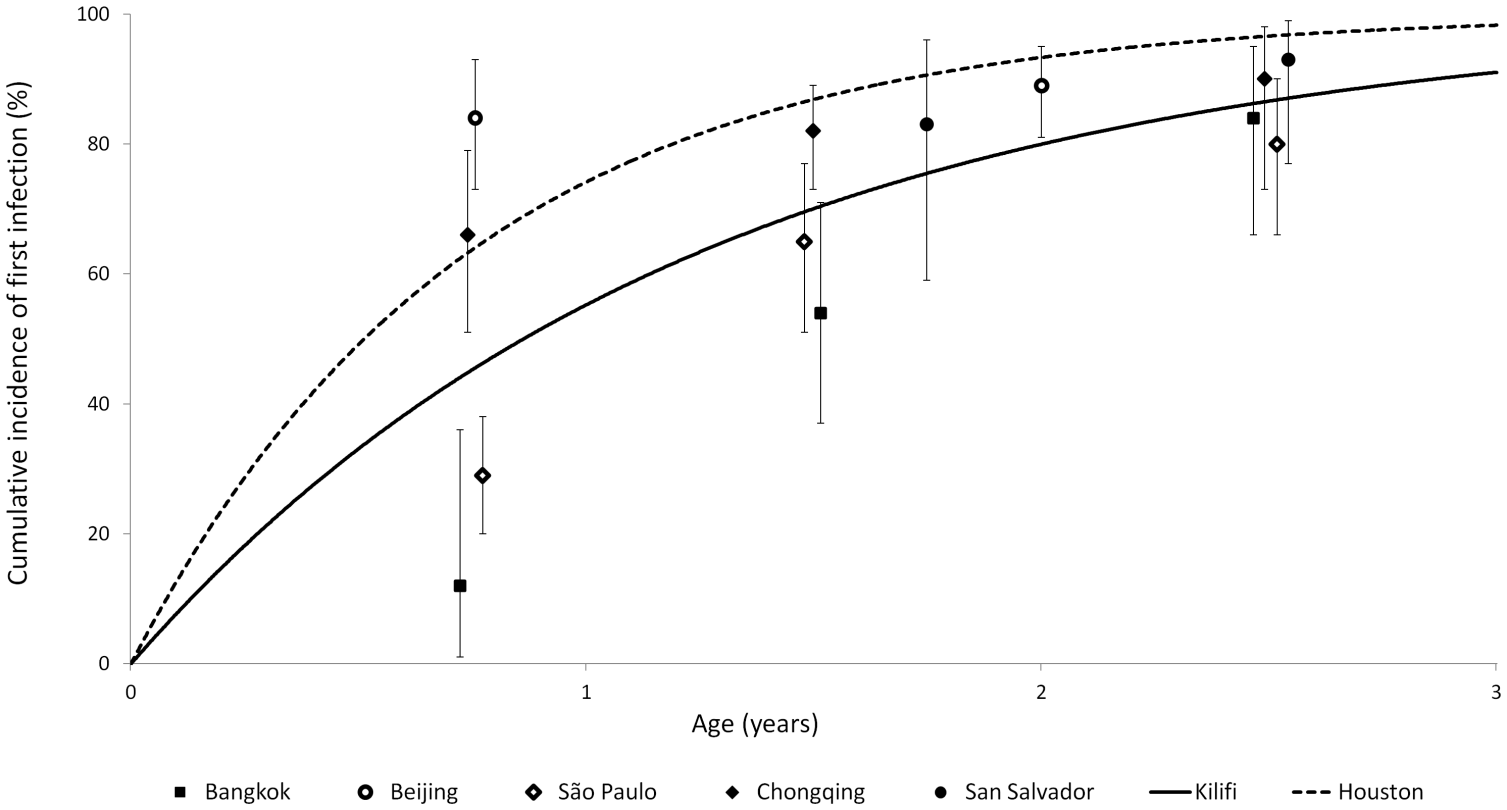
Cumulative incidence of first RSV infection according to age. Mean RSV incidence from birth cohorts in Kilifi and Houston (lines) compared to results from anti-RSV IgG seroprevalence surveys (data points with 95% CIs).

A number of studies have examined the seroprevalence of anti-RSV IgG antibodies according to age [16], [18], [19], [20], [21], [22]. Estimating the force of infection from these studies is problematic because of the highly seasonal nature of RSV infection (measuring seroprevalence just after the seasonal peak in RSV incidence will overestimate the average force of infection, while measuring seroprevalence just before the seasonal peak will underestimate the average force of infection). These studies suggest a relatively constant risk of RSV infection occurs over the first few years of life, with seroprevalence in the order of 90% by three years of age. In Figure 2 we compare the results of these seroprevalence studies with the RSV rates from the birth cohort studies from Kilifi and Houston. While the rate from the Kilifi study falls in the centre of the estimates from the seroprevalence studies, the rate from the Houston study appears to be higher than average.

We have used the rate of first RSV infections from the Kilifi study (0.0022 per day) as the estimate for mean λ for our model. In addition, in order to investigate how a higher than average risk of infection affects RSV seasonality, we also fitted the model using mean λ equal to the rate of first RSV infections from the Houston study (0.0037 per day).

#### B2. Susceptibility to reinfection

Previous infection with RSV provides incomplete immunity [23], [24], [25]. Natural immunity to RSV appears to have two aspects. Firstly, there appears to be a long term partial immunity following first infection, which we have modelled with a reduced susceptibility in the S_2_ category. This may be due to specific immunity following first RSV exposure, and/or the maturing of the infant immune system with age [16]. For the purposes of the model the distinction is not crucial. Secondly, there appears to be additional short term immunity following any RSV infection, which we have modelled by using the SEIRS (susceptible, exposed, infectious, recovered, susceptible) structure (as shown in Figure 1) rather than an SEIS structure. In the SEIRS model, individuals in the R category are resistant to infection, thus γ (the rate that these individuals transition from the R category to the S_2_ category) determines the duration of their short term immunity.

We estimated the susceptibility in the S_2_ category (α) as follows. It is generally accepted that susceptibility to first RSV infection is 100% (once protection from maternal antibody is lost). Infants in high exposure settings such as child care show RSV incidence approaching 100% during their first RSV season [26], [27]. Bearing this in mind, we considered that the attack rate following experimental infection of young adult volunteers should give an estimate of the susceptibility to infection in category S_2_. We identified four such studies [24], [28], [29], [30] (Table 2). Pooling the results of these studies gives α  = 0.77 (95% CI 0.68 to 0.84). We also found four observational studies in children where the relative risk of subsequent infection a year or more after first RSV infection was able to be calculated from the published data [16], [17], [26], [27] (Table 2). We pooled the results from these studies (weighted according to the number of children at risk of reinfection in each study) and the result (RR = 0.76) was consistent with the adult infection studies. In the model we use 0.68 to 0.84 for α.

**Table 2:** Data used to estimate susceptibility in S_2_ category in the model.

Setting	Study type	Susceptibility inS_2_ category
UK [29]	Experimental infection in adults	0.74 (0.49 to 0.91)^a^
USA [24]	Experimental infection in adults	0.76 (0.58 to 0.89)^a^
USA [28]	Experimental infection in adults	0.78 (0.62 to 0.89)^a^
USA [30]	Experimental infection in adults	0.77 (0.60 to 0.90)^a^
Kenya [16]	Observation study in children	0.93 (0.69 to 1.24)^b^
USA [17]	Observation study in children	0.71 (0.61 to 0.84)^b^
USA [26]	Observation study in children	0.72 (0.62 to 0.84)^b^
USA [27]	Observation study in children	0.67 (0.55 to 0.81)^b^

a Proportion (infected/challenged).

b RR (second infection/first infection, where second infection 12 or more months after first infection).

We estimated the rate of loss of short term immunity to reinfection (γ) as follows. We could not use data from observational studies to assess the short term risk of reinfection because these will be prone to bias due to the highly seasonal nature of RSV infection (as most people are infected with RSV during the seasonal epidemics, their risk from naturally occurring infection in the months following this will be low regardless of their degree of susceptibility). For this reason we have based our estimate of short term immunity on data from the single study investigating experimental reinfection of volunteers soon after confirmed natural RSV infection [23]. In this study 47% (95% CI 21% to 73%) of the subjects were infected upon experimental exposure to RSV two months after natural infection. We calculated γ by fitting a function:
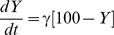



where *Y* is the population level susceptibility to reinfection, and t is the time following infection, to pass though the data point from this experimental study (47% susceptible at 2 months after previous infection) and then reach our estimate for α (77% susceptible at 12 or more months after first infection). This gave γ  = 0.016 per day. For the model we have used a range from 0.012 per day to 0.024 per day. The lower limit of the range is the lowest rate that will result in 100% of individuals having moved to category S_2_ within 1 year following infection. The upper limit of the range is 50% above the 0.016 estimate.

#### B3. Latent period (1/σ)

Studies of household RSV spread have found the serial interval to be approximately six to seven days [13], [14]. In four studies of experimental infection of adult volunteers, shedding was found to start at a mean of 6 days, 4.4 days, 4.4 days, and 4.0 days after inoculation (respective study sizes were 15, 30, 22 and 35) [23], [24], [28], [30]. In one of these studies it was noted that the latent period depended on the size of the infective dose, which may explain the differences between these three studies [24]. Standard texts estimate the mean incubation period to be 5 days [31]. The latent period is likely to be similar, as infectiousness will increase with the onset of symptoms, even if virus can be isolated prior to this [28]. In the model we use a mean latent period of 4 to 6 days. For the purpose of fitting the model to the observed RSV cases, we assume the latent period and incubation period are equal.

#### B4. Duration of infectiousness (1/ν_1_ and 1/ν_2_)

We have assumed the infectious period is the same as the duration of RSV shedding. Data from community studies are summarised in Table 3 (we excluded two studies measuring shedding in hospitalised children because more severe disease increases shedding duration [32], [33]). The Kenyan study summarised in Table 3 indicates that shedding duration varies according to the history of previous RSV infection [34]. In this study the mean duration of RSV shedding in those having had a previous RSV infection was 4.0 days, which is consistent with the results from four experimental infection studies in adults [23], [24], [30], [35]. Shedding appears longer in young children (approximately 5 to 6 days) consistent with the increased shedding duration during first RSV infection in the Kenyan study (5.1 days) [32], [34], [36]. In the model we use a mean duration of infectiousness of 5 to 6 days for individuals is category I_1_, and 4 days for individuals in category I_2_.

**Table 3:** Data on the duration of RSV shedding used to estimate the infectious period.

Setting	n	Age	Mean duration of RSVshedding
Kenya [34]	96	First infection	5.1 days (95% CI 4.2 to6.2 days)
	96	Previouslyinfected	4.0 days (95% CI 3.3 to4.9 days)
USA [24]	22	Adults	3.5 days
USA [23]	12	Adults	4.7 days
USA [35]	118	Adults	3.9 days
USA [30]	35	Adults	3.6 days
USA [36]	44	Less than 4 years	5 to 6 days
USA [32]	12	Less than 2 years	9.0 days (95% CI 2.0 to16.0 days)

#### B5. Relative degree of infectiousness between categories I_2_ and I_1_ (δ)

In a series of studies the degree of RSV shedding was found to be lower in adults (mean 3.0 log_10_TCID_50_/ml, n = 29) [23], [35], compared to hospitalised children aged less than 3 years (4.3 log_10_TCID_50_/ml, n = 19 ) [32]. This limited evidence suggests that individuals in category I_2_ are 0.7 times as infectious (in addition to having a shorter duration of infection) than those in category I_1_. A recently published RSV model with lifelong partial immunity following first infection used a relative degree of infectiousness between individuals in category I_2_ compared to category I_1_ of 0.6, and achieved good fitting to incidence data from a number of settings with varied seasonal patterns [37]. In the model we use 0.5 to 0.8 for δ.

### C. Model Fitting

We have fitted the incidence of I_1_ to the observed RSV cases, as the risk of severe disease and hospital admission is highest upon first infection [17], [26]. To fit the model to the observed monthly RSV cases, we first scaled up the cumulative incidence of I_1_ for each month to calculate the predicted monthly number of RSV cases (we derived the scaling factor from the mean number of observed RSV cases per year divided by the yearly cumulative incidence of I_1_ in the model at equilibrium). The seasonal forcing parameters ε, ψ and φ were derived from fitting the predicted number of RSV cases to the observed RSV cases, while at the same time fitting the model to the estimated average force of infection (mean λ) in the population. As the latent and incubation periods are the same in our model, the seasonal pattern of disease incidence will be coincident with the seasonal pattern of the incidence of I_1_. A formal least squares approach was used for model fitting. The parameter estimates used in the model are summarised in Table 1. Parameters were varied in a stepwise manner to maximise and minimise the time lag between the seasonal peak in β and the seasonal peak in RSV incidence, in order to form a range of epidemiologically plausible lag periods.

### D. Assessing Potential Drivers of RSV Seasonality

After using the model to estimate the seasonal pattern of β, we then compared this to the seasonal patterns of candidate drivers of RSV seasonality (meteorological factors, school holidays, and the seasonality of nutritional status). Meteorological factors examined were the number of days per week with rainfall >5 mm (chosen to incorporate both rainfall frequency and intensity) and mean relative humidity and mean temperature. In addition we estimated the dew point temperature (a measure of absolute humidity) from the mean relative humidity and mean temperature [38]. Meteorological data were recorded at the Philippine Atmospheric, Geophysical and Astronomical Services Administration (PAGASA) offices in Tagbilaran. All trial municipalities were within 25 km of Tagbilaran. The number of rainy days per week also serves as a proxy measure of sunshine hours, as these variables are negatively correlated in this tropical setting [7]. The seasonality of infant nutritional status was depicted using a time series of estimated birth weight in the PCV trial participants [6]. While birth weight is dependent on maternal nutrition, we have previously shown in our study setting that birth weights are lowest when rice production in lowest, and the seasonal trough in birth weight is coincident with a seasonal trough in infant growth [6]. Similarly, a study from Bangladesh found that the seasonality of birth weight was correlated both with nutrition in children aged less than five years, and with rice availability [39]. The seasonal scale variations of these environmental exposures were calculated using locally weighted regression scatter plot smoothing (lowess) using a five month bandwidth, before being compared to our estimate of the seasonal pattern of β.

## Results

**Figure 3:**
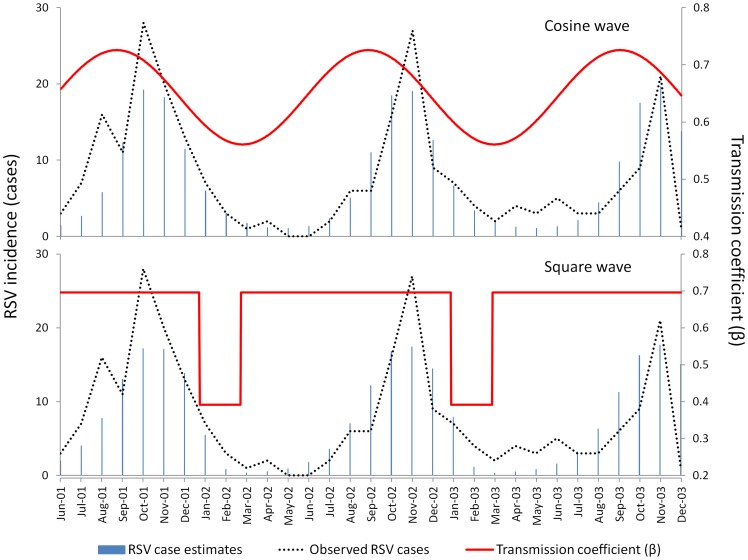
Results of the RSV model. RSV case estimates derived from the model were fitted to the observed number of RSV cases. Mean λ  = 0.0022 per day. Data from Bohol, the Philippines, 2001 to 2004.

Modelling the seasonality in births alone without additional seasonal variation in β could not reproduce the observed seasonality in RSV admissions, with the modelled incidence of RSV cases varying by less than 10% over the calendar year. Table 4 shows the results of the cosine β model, including the results for the parameter combinations resulting in the best model fit to the observed data, as well as the longest and shortest lag between the seasonal peak in β and the seasonal peak in RSV cases predicted by the model. While there is little difference in the model fit between the different parameter combinations (as demonstrated by the similarly sized residuals) the lag between the seasonal peak in β and the seasonal peak in RSV cases predicted by the model varies considerably. For the scenarios with a mean λ of 0.0022 this lag ranges from 67 days to 49 days. For the scenarios with a mean λ of 0.0037 (above average force of infection) the range of this lag is much wider, from 43 days to 6 days. Table 5 shows the results of the square wave β model, including the results for the parameter combinations resulting in the best model fit to the observed data, as well as the longest and shortest lag between the seasonal peak in RSV cases predicted by the model and the start of the square wave. The square wave β model does not fit the observed data as well as the cosine β model, as demonstrated by the larger residuals. For the scenarios with a mean λ of 0.0022 the lag between the seasonal peak in RSV cases predicted by the model and the start of the square wave ranges from 31 days to 68 days. For the scenarios with a mean λ of 0.0037 (above average force of infection) the range of this lag is from 80 days to 129 days. Figure 3 shows the cosine β and square wave β models fitted to the observed RSV cases, for the scenario with a mean λ of 0.0022 and the parameter combinations resulting in the best model fit to the observed data.

**Table 4:** Results from the cosine β model.

Mean λ		γ	α	σ	ν_1_	ν_2_	δ	ε	ψ	φ	Res	Lag
0.0022	Longest lag	0.024	0.68	0.17	0.17	0.25	0.5	0.72	0.20	140	24.9	67
	Best fit	0.012	0.84	0.25	0.17	0.25	0.5	0.64	0.13	123	24.6	51
	Shortest lag	0.012	0.84	0.25	0.17	0.25	0.8	0.42	0.13	122	24.6	49
0.0037	Longest lag	0.024	0.68	0.17	0.17	0.25	0.5	0.78	0.14	116	26.0	43
	Best fit	0.012	0.84	0.25	0.17	0.25	0.8	0.47	0.12	81	24.1	9
	Shortest lag	0.012	0.84	0.25	0.17	0.25	0.5	0.72	0.11	77	24.4	6

The lag is from the seasonal peak in β to the seasonal peak in RSV cases predicted by the model. Lag and φ are in days. The values of λ, γ, σ, ν_1_ and ν_2_ are rates per day. Res is the residuals following fitting the model to the observed RSV data (the square root of the sum of the squares of the difference between the monthly number of observed RSV cases and the monthly number of RSV cases predicted by the model).

**Table 5:** Results from the square wave β model.

Mean λ		γ	α	σ	ν_1_	ν_2_	δ	ε	ψ	φ	Res	Lag
0.0022	Shortest lag	0.024	0.68	0.17	0.17	0.25	0.5	0.72	0.48	164	29.3	31
	Best fit/longest lag	0.012	0.84	0.25	0.17	0.25	0.5	0.65	0.47	156	26.9	68
0.0037	Shortest lag	0.024	0.68	0.17	0.17	0.25	0.5	0.79	0.55	143	28.5	80
	Best fit/longest lag	0.012	0.84	0.25	0.17	0.25	0.5	0.73	0.51	100	25.7	129

The lag is from the seasonal peak in RSV cases predicted by the model to the start of the square wave. Lag and φ are in days. The values of λ, γ, σ, ν_1_ and ν_2_ are rates per day. Res is the residuals following fitting the model to the observed RSV data (the square root of the sum of the squares of the difference between the monthly number of observed RSV cases and the monthly number of RSV cases predicted by the model).

**Figure 4:**
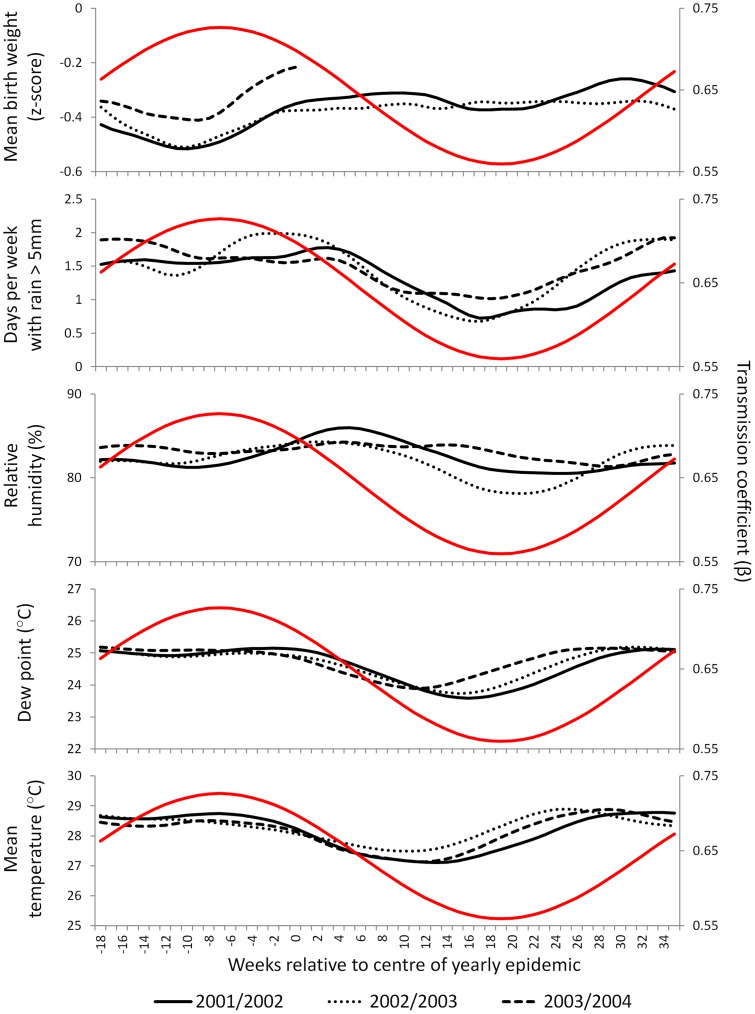
Timing of the estimated seasonal variation in the transmission coefficient (β) relative to observed seasonal exposures. The red line shows the estimated variation in β. The black lines show the variation in the observed exposures. Data from Bohol, the Philippines, 2001 to 2004.

Figure 4 shows the seasonal pattern of β estimated from the cosine β model with mean λ of 0.0022, compared to the seasonal scale variations in the potential environmental drivers. The environmental data are plotted relative to the centre of the yearly RSV epidemics, which occurred at slightly different times each year. The nutrition index (estimated weight for age z-score at birth) is consistently at its minimum 10 weeks before the centre of the yearly epidemics, compared to the seasonal peak in β, which occurred 7 weeks (51 days) before the peak in RSV cases. Although rainfall does not show a consistent seasonal peak, the seasonal trough in rainfall occurs consistently 17 to 18 weeks after the centre of the yearly RSV epidemics, compared to the seasonal trough in β, which occurred 19 weeks (131 days) after the peak in RSV cases. In contrast, relative humidity, dew point and temperature do not show clear relationships with the seasonal pattern of β. Performing a similar analysis for school terms indicates that school holidays begin after the predicted square wave. This can be seen in Figure 3 (summer school holidays in the Philippines begin in late March to early April).

## Discussion

We have found that the observed seasonality of RSV admissions in our study setting can be replicated by an RSV transmission model with a transmission coefficient (β) following a seasonal cosine wave that is maximal 51 days (range 67 days to 49 days) before the yearly peak in RSV admissions. This is consistent with the findings from previous modelling of RSV infection, which suggests that peak RSV transmissibility precedes yearly RSV epidemics by one to three months [9]. Seasonal malnutrition in our study setting (as measured by seasonal variation in birth weight and infant growth) is consistently at its worst 10 weeks before the yearly RSV epidemics [6]. This lag is slightly longer than the 51 days predicted by the model, however there are three mechanisms we have not modelled which would lead to an additional delay between peak seasonal malnutrition and peak RSV admissions. Firstly, we have not factored in health care seeking delays: the children with RSV in our study had an average delay of 5 days between the onset of cough and admission. Secondly, the immune effects of malnutrition may lag behind the anthropometric effects: studies of the treatment of malnutrition in children indicate that immune recovery lags up to a month behind anthropometric recovery [40], [41], [42]. Thirdly, not all children suffer from malnutrition in our study setting, thus successive generations of infection are required before seasonal forcing in the malnourished subgroup could drive RSV incidence in the wider population. Rainfall is high most of the year in our study setting, and shows a consistent trough 17 to 18 weeks after the yearly RSV epidemics. This is also consistent with our model, which predicts the seasonal trough in β occurring 131 days (19 weeks) after the yearly peak in RSV cases. Factoring the health care seeking delay discussed above would mean the seasonal trough in β actually occurs 18 weeks after the yearly peak in RSV cases, precisely coincident with the observed trough in rainfall. The seasonal patterns of temperature, dew point and relative humidity are not consistent with the predicted seasonal pattern of β. The small magnitude of the seasonal variation in these exposures (mean temperature and dew point vary by approximately 2°C through the year, while mean relative humidity varies by approximately 5% through the year) also suggests that these exposures are unlikely to be playing a major role in driving the seasonality of RSV in this setting. School summer holidays appear to occur too late to play a dominant role in driving RSV incidence in infants in this setting. In addition, the timing of school holidays relative to yearly RSV epidemics is very different in other tropical climates, suggesting school holidays do not play a dominant role in RSV seasonality in the tropics. For example in northern Australia and in southeast Florida RSV incidence is highest soon after school summer holidays [43], [44].

Seasonal malnutrition is a biologically plausible driver of RSV seasonality: poor infant growth leaves children weakened and vulnerable to infection [45], and is a known risk factor for RSV associated ALRI [46], [47], [48], [49]. If rainfall is driving RSV transmission in this setting, the mechanism of action is less clear. Although high rainfall is linked to high humidity, our results suggest that changes in humidity are not the principle mechanism of action. It is hypothesised that adverse weather conditions such as increased rainfall could lead to increased indoor crowding, increasing respiratory virus transmission [5], [50]. There are little empirical data available to test this hypothesis. One study from the tropical setting of Bangladesh found an increased risk of respiratory infection following rainy days only occurred in households with three or more people per room, suggesting that the increased risk of RSV infection in crowded households is more pronounced during rainy days [51]. Cloud cover is increased during the rainy season. In tropical latitudes the amount of sunshine is dictated more by cloud cover than day length, with the lowest amount of sunshine during the rainy season. Low levels of 25-hydroxyvitamin D appear to be a risk factor for ALRI due to RSV in infants [52]. If children have higher vitamin D levels during the dry, sunny season this could reduce transmissibility by increasing innate defences in susceptible children. Whether vitamin D deficiency occurs in children in this tropical setting is unknown, as is the timing of any seasonal deficiency (in temperate settings, variations in vitamin D levels appear to lag 1 to 2 months behind variations in sunshine levels [53], [54], however the temporal relationship may be different in the tropics). These questions could be addressed directly by studies measuring the seasonal variation in Vitamin D levels in children in tropical settings. Increased sunshine may also act to directly kill pathogens in the environment [5].

It is possible that seasonal variations in both nutrition and rainfall could act synergistically to drive RSV transmission in this setting. According to our model, either exposure could potentially drive seasonality alone; however it is not clear if variations in either nutrition of rainfall patterns are large enough to drive the transmission coefficient to the necessary amplitude. If more than one mechanism was acting, each need only be responsible for part of the variation in the transmission coefficient.

Our model is based on a previous RSV model developed by Weber et al [9], however our model differs in several aspects. A central issue with RSV models is modelling partial immunity to reinfection. The previous model by Weber et al used stepwise reductions in susceptibility to reinfection following first, second and later infection cycles. In our model, after the first RSV infection, individuals have the same (reduced) susceptibility to any subsequent infections. Individual level studies examining this have not found a significant difference between the susceptibility to second and third RSV infection, indicating that our simplification is reasonable [16], [17], [26]. In addition, susceptibility to RSV infection appears similar in adults who have had many previous RSV infections, as well as in children who have had only one previous RSV infection (Table 2). Our second simplification to the Weber model is we do not include an epidemiological compartment for infants with maternal anti-RSV antibodies (i.e. all infants are born fully susceptible). This simplification has been used previously [37], [55]. Some of our parameter estimates differ from those used by Weber et al, in particular our estimates for the rate of loss of short term immunity (γ) and the duration of infectiousness (ν_1_ and ν_2_). In our model we chose a minimum value of γ  = 0.012 per day, compared to γ  = 0.005 per day in Weber et al. We limited γ to values above 0.012 per day as this is the lowest rate that will result in all infected subjects losing their partial immunity by one year following infection. This appears to be a reasonable assumption: data from Kenya and the USA suggest short term immunity lasts no more than a year [16], [17]. In contrast to our estimates for the duration of infectiousness (5 to 6 days for first infections and 4 days for later infections), Weber at al used 10 days. Our estimates were based on data from 7 studies examining RSV shedding, the two largest of which were published after the Weber model was developed. In addition, we specifically excluded studies examining RSV shedding in hospitalised children, as shedding duration is prolonged in children with RSV associated ALRI [32], [33], who make up only a small fraction the RSV cases occurring in the general population.

Our results show that the lag between the seasonal peak in β and the seasonal peak in RSV cases is substantially shorter if the mean force of infection (λ) is higher. We have used the average rate of first RSV infection in Kilifi (0.0022 per day) [16] as the mean force of infection for our primary analysis, while we have used the average rate of first RSV infection in Houston (0.0037 per day) [17] to give an indication of the effect of a particularly high force of infection. We used the Kilifi data to derive our primary force of infection estimate for two reasons. Firstly, Kilifi is similar to our study setting of Bohol in that both are rural, coastal settings in the tropics. Secondly, the average rate of first infection in Kilifi appears to be more consistent with the results from the seroprevalence studies in the literature than the rate of first infection in Houston (Figure 2). The authors of the Houston paper note that the cumulative incidence of infection in their study was similar to that found in child care settings in other similar studies, which suggests a particularly high risk of infection over the two years of the Houston study. While using the average rate of first RSV infection in Kilifi as the mean force of infection for our primary analysis appears valid, it is clear that for the best precision when estimating the seasonal pattern of β, precise measurement of the local force of infection is desirable.

In Figure 4 we plotted the environmental data relative to the centre of the actual yearly RSV epidemics, because epidemics occurred at slightly different times each year (the 2002/2003 epidemic occurred 56 weeks after the 2001/2002 epidemic, while the 2003/2004 epidemic occurred 50 weeks after the 2002/2003 epidemic). In doing so we have implicitly assumed year to year differences in the timing of the epidemics are due to differences in the timing of seasonal drivers, rather than due to differences from year to year in the amplitude of the seasonal forcing. However, as discussed in the previous paragraph, a higher force of infection is associated with a shorter lag between the seasonal peak in β and the seasonal peak in RSV cases. Thus a year with particularly intense seasonal increase in β would be expected to have an earlier RSV epidemic. Further modelling incorporating year to year differences in the amplitude of seasonal forcing may shed additional light on seasonality, particularly if modelling incidence during extreme rainfall events, or during poor harvests.

Our findings contribute to the current epidemiologic understanding of RSV, the most common cause of ALRI in infants and children [56]. We have shown that seasonal scale drivers of transmissibility can act several weeks before and/or after the seasonal peaks in RSV incidence, and thus it is important to incorporate the non-linear dynamics of infectious disease transmission when examining potential drivers of infectious disease seasonality. Our results indicate that the timing of seasonal variations in nutrition and rainfall patterns in this tropical study setting are epidemiologically consistent with them being plausible drivers of RSV seasonality.
